# Necrotic and Cytolytic Activity on Grapevine Leaves Produced by Nep1-Like Proteins of *Diplodia seriata*

**DOI:** 10.3389/fpls.2019.01282

**Published:** 2019-10-17

**Authors:** Rebeca Cobos, Carla Calvo-Peña, José Manuel Álvarez-Pérez, Ana Ibáñez, Alba Diez-Galán, Sandra González-García, Penélope García-Angulo, Jose Luis Acebes, Juan José R. Coque

**Affiliations:** ^1^Instituto de Investigación de la Viña y el Vino, Universidad de León, León, Spain; ^2^RGA-bioinvestigación S.L., León, Spain

**Keywords:** Botryosphaeriaceae, NLP, RT-qPCR, grapevine trunk diseases, phytotoxicity, *Vitis vinifera*

## Abstract

Many phytopathogenic fungi produce necrosis and ethylene inducing peptide 1 (Nep1-like proteins or NLP) that trigger leaf necrosis and the activation of defense mechanisms. These proteins have been widely studied in plant pathogens as *Moniliophthora perniciosa* or *Botrytis cinerea* between others, but little is known about their biological roles in grapevine trunk pathogens. Advances in the sequencing of genomes of several fungi involved in grapevine trunk diseases have revealed that these proteins are present in several copies in their genomes. The aim of this project was to analyze the presence of genes encoding NLP proteins in the *Diplodia seriata* genome and to characterize their putative role as virulence factors associated to grapevine trunk diseases. In this study, we characterized four NLPs from *Diplodia seriata*. All proteins showed highly similar amino acid sequences and contained the characteristic peptide motifs of NLPs. DserNEPs slightly reduced the viability of *Vitis vinifera* L. cell cultures. The cytolytic activity from DserNEP1 was stronger than that from DserNEP2, even at low concentrations. Purified DserNEPs also produced necrosis in leaves when they were inoculated into micropropagules of *V. vinifera* L. This is the first record of Nep1-like proteins from a fungus associated with grapevine trunk diseases and also from a member of the Botryosphaeriaceae family.

## Introduction

Grapevines are one of the most important economic crops worldwide. Grapevine trunk diseases (GTDs) are a major threat for the wine sector, causing serious economic losses to the wine industry (Siebert, 2001; Gubler et al., 2005). This term encompasses different fungal pathologies such as Botryosphaeria dieback, Esca, Eutypa dieback, Petri disease, or Black foot, to cite the most relevant. The incidence of GTD have increased over the last decades, mainly due to the lack of effective strategies to fight these diseases (Chiarappa, 2000; Graniti et al., 2000; Spagnolo et al., 2017; Mondello et al., 2018). Botryosphaeria dieback has been reported since the 1970s as one of the main GTD. It is caused by several xylem-inhabiting fungi (Bertsch et al., 2013) which are primarily members of the Botryosphaeriaceae family such as *Diplodia seriata* De Not. (anamorph of *Botryosphaeria obtusa*, Shoemaker, 1964; Phillips et al., 2007), *Diplodia mutila* (anamorph of *Botryosphaeria stevensii,* Shoemaker, 1964), and *Neofusicoccum parvum* (anamorph of *Botryosphaeria parva*, Crous et al., 2006).

*D. seriata* is one of the pathogens most frequently isolated from symptomatic grapevines in Spanish vineyards (Martín and Cobos, 2007) as well as French vineyards (Kuntzmann et al., 2010). It has been isolated from at least 34 different hosts (Punithalingam and Waller, 1976), mostly fruit trees, but also from other woody plants. *D. seriata* has been recognized as a wound pathogen and is associated with dieback symptoms and cankers (Larignon et al., 2001; Phillips, 2002; Van Niekerk et al., 2004). These pathogens attack the perennial organs of grapevines, although they have never been isolated from leaves (Larignon and Dubos, 1997). Therefore, it is supposed that symptoms observed in the berries and leaves might be caused by extracellular compounds produced by the fungi in colorless woody tissues of the trunk, which are then translocated to leaves through the transpiration system (Mugnai et al., 1999).

The toxicity of some extracellular metabolites produced by members of Botryosphaeriaceae family has been proven (Martos et al., 2008; Andolfi et al., 2011; Ramírez-Suero et al., 2014), and several phytotoxic metabolites have been identified (Bénard-Gellon et al., 2014; Abou-Mansour et al., 2015). However, little is known about putative proteins secreted by these fungi that could have any role in pathogenesis. Bénard-Gellon et al. (2014) detected some toxicity in an extracellular protein extract from *D. seriata* and *N. parvum*. However, to date no protein has been identified yet as a virulence factor in *D. seriata*, although in a previous work Nep1-like proteins were detected as putative virulence factors in the secretome of this fungus (Cobos et al., 2010). Among the host cell-death-inducing secreted proteins of plant pathogens are the necrosis and ethylene-inducing peptide 1 (Nep1)-like proteins (NLPs) that were first detected in culture filtrates of *Fusarium oxysporum* (Bailey, 1995). NLPs constitute a superfamily of proteins that are produced by phytopathogenic bacteria, fungi, and oomycetes (Pemberton and Salmond, 2004; Gijzen and Nurnberger, 2006), which have been recognized as virulence factors in several phytopathogenic fungi such as *F. oxysporium* (Bailey, 1995; Bae et al., 2006), *Botrytis* spp. (Staats et al., 2007a; Staats et al., 2007b; Schouten et al., 2008), *Phytophthora megakarya* (Bae et al., 2005), *Moniliophthora perniciosa* (García et al., 2007; Zaparoli et al., 2009), *Mycosphaerella graminicola* (Motteram et al., 2009), or *Verticillium dahliae* (Zhou et al., 2012) among others.

NLPs have been proposed to have dual functions in plant–pathogen interactions, acting both as triggers of immune responses and also as toxin-like virulence factors (Qutob et al., 2006). NLPs are relatively small proteins of about 24 kDa that exhibit a high degree of similarity at amino acid sequence level, including the presence of two highly conserved cysteine residues that form an intramolecular disulfide bridge essential for NLP activities (Fellbrich et al., 2002; Qutob et al., 2006; Ottmann et al., 2009), and also a central hepta-peptide motif “GHRHDWE” that is part of the negatively charged cavity exposed at the protein surface. Both are necessary for plasma membrane permeabilization and cytolysis in plant cells (Ottmann et al., 2009). NLPs have been classified into type I and type II classes, depending on whether they contain two or four cysteine residues present at conserved positions, respectively (Gijzen and Nurnberger, 2006). A third type of NLP was described by Oome and Van Den Ackerveken (2014), but this type III only shares a central 50 amino acids with types I and II including the highly conserved heptapeptide motif.

The present study aims to analyze the presence of genes encoding NLP proteins in the *D. seriata* genome and to characterize their structure and their putative role as virulence factors associated to GTDs.

## Materials and Methods

### DNA Isolation

The *D. seriata* strain used in this work was derived from a monosporic culture of *D. seriata* VS1 (Cobos et al., 2010). The strain was routinely maintained on potato dextrose agar (PDA; Scharlau Chemie S.A.). Genomic DNA was isolated from fresh mycelia following an adaptation of the method described by Möller et al. (1992). Briefly, 16 g of fresh mycelia was ground to a fine powder in liquid nitrogen, transferred to Falcon® tubes, and mixed with TES solution (100 mM Tris, pH 8.0, 10 mM EDTA, 2% SDS); 120 µg/mL of Proteinase K was added and tubes were incubated for 1 h at 65°C with occasional gentle mixing. The salt solution was adjusted to 1.4 M with 5 M NaCl, and 1/10 volume 10% CTAB was added before incubating for 10 min at 65°C. The tubes were centrifuged at 10,000 rpm for 10 min. RNase (20 µg/mL) was added to the supernatant and incubated at 37°C for 1 h. The aqueous phase was extracted with 1 volume of phenol–CIA solution (phenol/chloroform/isoamyl alcohol; 25:24:1 v/v) mixed by inversion and placed on ice for 30 min. After centrifugation for 10 min at 8,000 rpm, and an additional extraction with 1 volume of CIA (chloroform:isoamyl alcohol; 24:1 v/v), the supernatant was mixed with 1/3 volume of 5 M NH4Ac, mixed gently, and placed on ice for 30 min. After centrifugation for 10 min at 8,000 rpm, the nucleic acids were precipitated with 1 volume of cold isopropanol. DNA was recovered by centrifugation and the pellet washed with 70% ethanol. DNA was dissolved in 500 µl of TE buffer and stored at −20°C. DNA concentration was estimated with a NanoDrop 2000 Spectrophotometer (Thermo Scientific).

### Genomic Library Construction and Screening

Genomic DNA from *D. seriata* (12 µg) was partially digested with *Sau*3AI. DNA fragments (17–23 kb) were purified by ultracentrifugation in a sucrose gradient and ligated to Lambda DASH II *Bam*HI Vector Kit (Stratagene), followed by *in vitro* packaging. Degenerate primers NepFdeg (5′ GTRAATGGRTGCGTRCCATTCCC 3′) and NepRdeg (5′ CCTTCCCARTCGTGRCGGTGRCC 3′) were designed against conserved regions present in Nep1-like proteins (identified by *in silico* analysis of proteins deposited in GenBank database) that included the peptides previously identified by MASCOT from *D. seriata* NLPs (Cobos et al., 2010). These primers amplified a partial DserNEP sequence that was labeled with the DIG DNA labeling kit (Roche) and used as a hybridization probe to screen recombinant bacteriophage plaques of the genomic library.

### PCR Amplification and Sequencing of *DserNEP* Genes

Primer pairs were designed to amplify the entire sequence of *DserNEP* genes from DNA and cDNA (**Table 1**). *DserNEP* genes were amplified by PCR. Each reaction contained 1× Kapa Hifi (KAPA BIOSYSTEMS), 300 µM of each dNTP, 0.3 µM of each primer, 0.5 U of Kapa Hifi polymerase, and 1 µl of template DNA. PCR amplifications were performed on a Mastercycler gradient (Eppendorf). The program consisted of an initial step of 2 min at 95°C, followed by 35 cycles of denaturation at 95°C for 20 s, annealing at 60°C for 15 s, and elongation at 72°C for 30 s. A final extension was performed at 72°C for 3 min. DNA was sequenced by the dideoxynucleotide chain termination method using a BigDye Terminator cycle sequencing kit (Applied Biosystems). Signal peptide regions were predicted by using the Signal P3 program (Bendtsen et al., 2004).

**Table 1:** Oligonucleotides used in this study.

Primer name	Primer sequence (5′–3′)
**NepFdeg**	GTRAATGGRTGCGTRCCATTCCC
**NepRdeg**	CCTTCCCARTCGTGRCGGTGRCC
**DserNEP1F**	ATGCTGTCCTCATCACTCTTCTGGCC
**DserNEP1R**	TCACAACGCAGCCTCAGCGAGGTT
**DserNEP2F**	ATGCCGCTCTCCATCCGCTAC
**DserNEP2R**	TCACAACGCCGCCTTGGCCAG
**DserNEP3F**	GCCCCCTTCACCCAGCAGCTGCACG
**DserNEP3R**	TCAAACCCACGCCTTATCCAAATTCCCC
**DserNEP4F**	GCCCCGGCAGCTGCCCCTGAGAG
**DserNEP4R**	TCAAAGGCAGGCCTTGTTAAGGTTG
**qDsernep1F**	ACGCTTTCGCCATCATGTAC
**qDsernep1R**	ACAATGCTCTCCCAGTCGTG
**qDsernep2F**	TACAACGTCTACCCCGTCAAC
**qDsernep2R**	TCTTTGAACGGCACATTGGC
**qDsernep3F**	GGTATGCGTTGCTGGATTGGGATGT
**qDsernep4F**	CGAGCTGCAGTTCAAGACCAGC
**qtubulin Dser F**	GAACGTCTACTTCAACGAGGT
**qtubulin Dser R**	GAGGACAGCACGAGGAACGT

### Amino Acid Sequence Analysis

Sequences from GTD pathogens with significant similarity (value 1e^−4^) to DserNEP proteins were retrieved from the NCBI database and identified using BlastP (Altschul et al., 1990). Sequences in **Table 2** were aligned by ClustalW (Larkin et al., 2007) and then analyzed using the MEGA 5 phylogenetic package (Tamura et al., 2011). The phylogenetic tree was obtained using Neighbor analysis with 1,000 bootstrap replications.

**Table 2:** Nep 1-like proteins used in the phylogenetical analysis.

Organism	Accession number	Plant pathogenicity of host	Reference
***Diplodia seriata***	KKY26562	Botryosphaeria dieback	Morales-Cruz et al., 2015
***Diplodia seriata***	KKY20654	Botryosphaeria dieback	Morales-Cruz et al., 2015
***Diplodia seriata***	KKY13781	Botryosphaeria dieback	Morales-Cruz et al., 2015
***Diplodia seriata***	KKY20647	Botryosphaeria dieback	Morales-Cruz et al., 2015
***Diplodia seriata***	AKQ49205	Botryosphaeria dieback	This study
***Diplodia seriata***	AKQ49206	Botryosphaeria dieback	This study
***Diplodia seriata***	MK978328	Botryosphaeria dieback	This study
***Diplodia seriata***	MK978329	Botryosphaeria dieback	This study
***Neofusicoccum parvum***	EOD44475	Botryosphaeria dieback	Blanco-Ulate et al., 2013b
***Neofusicoccum parvum***	EOD47698	Botryosphaeria dieback	Blanco-Ulate et al., 2013b
***Neofusicoccum parvum***	EOD48844	Botryosphaeria dieback	Blanco-Ulate et al., 2013b
***Neofusicoccum parvum***	EOD52252	Botryosphaeria dieback	Blanco-Ulate et al., 2013b
***Neofusicoccum parvum***	EOD44269	Botryosphaeria dieback	Blanco-Ulate et al., 2013b
***Phaeoacremonium minimum***	XP007911059	Esca disease	Blanco-Ulate et al., 2013c
***Eutypa lata***	EMR66076	Eutypa dieback	Blanco-Ulate et al., 2013a
***Eutypa lata***	EMR63075	Eutypa dieback	Blanco-Ulate et al., 2013a
***Eutypa lata***	EMR70921	Eutypa dieback	Blanco-Ulate et al., 2013a
***Diaporthe ampelina***	KKY35834	Phomopsis dieback	Morales-Cruz et al., 2015
***Diaporthe ampelina***	KKY36076	Phomopsis dieback	Morales-Cruz et al., 2015
***Diaporthe ampelina***	KKY37779	Phomopsis dieback	Morales-Cruz et al., 2015
***Diaporthe ampelina***	KKY30513	Phomopsis dieback	Morales-Cruz et al., 2015

### Analysis of DserNLP Expression

A plug of mycelium of *D. seriata* VS1c grown on PDA was inoculated in Erlenmeyer flasks containing Czapeck liquid medium (control conditions) (Cobos et al., 2010) or Czapeck liquid medium supplemented with chips of grapevine wood as described by Paolinelli-Alfonso et al. (2016). Each condition was assayed in triplicate. After inoculation, flasks were incubated in an orbital shaker at 25°C and 100 rpm in darkness. Fungal mycelia were collected from each 24 hours during 6 days. All collected samples were immediately frozen in liquid nitrogen and stored at −70°C for RNA isolation. Total RNA was isolated with 1 mL of TRIzol reagent (Invitrogen) according to the manufacturer’s instructions. RNA was cleaned up with the RNeasy Plant Mini Kit (Qiagen) including the on-column DNase enzymatic treatment. RNA concentration and purity were measured with NanoDrop 2000 Spectrophotometer (Thermo Scientific). cDNA was synthesized from RNA with PrimeScript RT Master Mix (Takara). Transcript levels of NLPs were determined by quantitative real-time PCR (qRT-PCR, TB Green Premix Ex Taq; Takara). qRT-PCR reactions were carried out in triplicate in 96-well plates in a 20-µl final volume containing 1× TB Green Premix Ex Taq (Takara), and 400 nM forward and reverse primers (**Table 1**). Cycling parameters were 2 min of Taq polymerase activation at 95°C, followed by 40 two-step cycles composed of 20 s of denaturation at 95°C, and 20 s of annealing and elongation at 58°C. Melting curve assays were performed from 55 to 95°C, and melting peaks were visualized to check the specificity of amplification. The results obtained for each gene of interest were normalized to the expression of β-tubulin gene. Three biological with three technical replicates were used. Relative gene expression was determined with the formula fold induction: 2^−ΔΔCt^, where ΔΔCt = [Ct TG (US) – Ct RG (US)] – [Ct TG (RS) – Ct RG (RS)]. Ct (cycle threshold) value is based on the threshold crossing point of individual fluorescence traces of each sample, TG is target gene, RG is reference gene, US is unknown sample, and RS is reference sample. The genes analyzed were considered significantly up- or downregulated when changes in their expression were >2-fold or <0.5-fold, respectively.

### Heterologous Expression in *Escherichia coli*

The cDNAs encoding for DserNEP1 and DserNEP2 proteins (without their putative signal peptides) were amplified and cloned into pET SUMO vectors and transformed into competent *E. coli* One shot Mach1-T1 cells. Recombinant clones were selected and sequenced to ensure that no erroneous nucleotide changes had resulted from PCR amplification. Expression of recombinant proteins in *E. coli* BL21 (DE3) strain was carried out by using the Champion pET SUMO Protein Expression System (Invitrogen) according to the manufacturer’s instructions. Purification of His-tag fusion proteins from *E. coli* cell-free extracts was achieved by affinity purification with a Ni-nitrilotriacetic acid resin (Ni-NTA; Qiagen) balanced with buffer A (50 mM NaH_2_PO_4_; 300 mM NaCl; 20 mM imidazole; pH 8.0). After extensive washing, bound proteins were eluted with buffer B (50 mM NaH_2_PO_4_; 300 mM NaCl; 250 mM imidazole; pH8.0). Upon visual inspection in a SDS gel, DserNEP-containing fractions were pooled and dialyzed against a phosphate-buffered saline (PBS) solution (pH 7.4) at 4°C with a Slide-A-lyzer Mini Dialysis Float system (Pierce). The purity of the recombinant proteins (higher that 98%) was confirmed by SDS PAGE and quantified using the Bradford method (Bradford, 1976).

### Grapevine *In Vitro* Cultures and Cellular Callus Production

For the development of *in vitro* cultures, grapevine shoots of Tempranillo cultivar, with three to four buds each, were treated with 16% (w/v) copper oxychloride fungicide (Cobre Key-S; Químicas KEY S.A.). Buds were stimulated to sprout under culture room conditions maintained at 25 ± 2°C with a 16/8-hour light/dark cycle for 2 months. Two kinds of explants were used: double-node stem segments for *in vitro* plants and young leaves for calluses. Explants were surface sterilized by immersion in 70% (v/v) ethanol for 1 min, and 0.4% (v/v) sodium hypochlorite solution with four drops of Tween 20 for 2 min, and then rinsed four to five times in sterilized water.

*In vitro* plants were obtained according to Sevillano et al. (2014). Double-node stem segment explants were cultured on Murashige and Skoog media (Murashige and Skoog, 1962) supplemented with 20 g/L sucrose, 1 mg/L benzyl adenine (BA), and 8 g/L agar, pH 5.8. Stems developed from nodal segments were multiplied through micro-cutting in order to obtain *in vitro* plants. All plant material was grown at 25 ± 2°C under 16/8-hour light/dark cycle and transferred to fresh medium every 2 months.

For callus induction, young sterilized grape leaves from Tempranillo cultivar were cultured on GB5 media (Gamborg et al., 1968) supplemented with 20 g/L sucrose, 1 mg/L 2,4-dichlophenoxyacetic acid (2,4-D), 0.1 mg/L BA, 0.5% (w/v) charcoal, and 8 g/L agar, pH 5.8. Calluses were maintained at 25 ± 2°C in darkness and transferred to fresh medium monthly. For establishment of liquid cell suspensions, 1 g of callus pieces was transferred into 150 mL flasks containing 50 mL liquid GB5 media supplemented with 20 g/L sucrose, 0.5 mg/L 2,4-D pH 5.8, placed in a rotary shaker (120 rpm) under 16/8-hour light/dark cycle, and routinely subcultured every 15 days. Manipulation of plant material was always performed on a clean bench and all instruments and growth media used were sterilized using dry heat or in autoclave.

In order to test the cultivar susceptibility, 1-year-old plants of four different cultivars (Chardonnay, Cabernet Sauvignon, Tempranillo, and Sauvignon Blanc) were potted in plastic pots and regularly irrigated by drip.

### Necrosis Activity Assay

*In vitro* micropropagated *V. vinifera* plants from Tempranillo cultivar were inoculated with purified DserNEP1, DserNEP2, or PBS buffer (pH 7.4). We tested different protein concentrations ranging between 0 and 0.5 mg/mL. Protein application was made by dipping freshly cut micropropagules into DserNEP protein solution (100 µl), and the propagules were immediately transferred to fresh medium. Each concentration was assayed in three replicates and the experiment was repeated at least three times.

Application to potted plants was carried out by leaf infiltration of 20 µl of DserNEP1 and DserNEP2 proteins at 0.15 and 0.30 mg/mL, or PBS buffer (as negative control). Three plants for each cultivar and three leaves from each plant were assayed.

Necrosis quantification was carried out by performing an electrolyte leakage assay, as described previously (Sevillano et al., 2014), by using 50 mg of leaves that were removed and washed in 2 mL distilled water 4 days after propagule inoculation. After 5 min, the water was transferred to other tubes and electrolyte leakage was measured with a conductivity meter (Crison 522).

### Fluorescein Diacetate Assay (FDA)

FDA assay was used to check cell viability. FDA is converted by non-specific esterases of vital cells to fluorescein, which produces a bright green fluorescence for at least 15 min. The polar fluorescein is trapped in cells with intact plasma membranes (Widholm, 1972). Cell suspensions were exposed to purified DserNEP1 (0.15 mg/mL) and DserNEP2 (0.60 mg/mL). Each concentration was assayed in triplicate. Cell viability was measured by staining with FDA [1:1 (v/v) dilution of 0.1 mg/mL FDA] 5 days after inoculation. After 2 min in darkness, cells were observed under a Nikon microscope equipped with epifluorescence irradiation. Cell viability was expressed as the percentage of fluorescent cells with respect the total number of cells checked. The experiment was repeated three times.

### Statistical Data Analysis

Conductivity and cell viability data were analyzed using a weighted least-square ANOVA test to determine if there were significant differences. When *F* ratios were statistically significant, *post hoc* tests (Tukey’s honestly significant difference test) were performed to establish where the differences between groups were. Statistical analyses were performed using R Core Team 3.0.1 (2014) software (http://www.R-project.org). Error bars in graphs indicate SDs. Bars marked with the same letter do not differ at P = 0.05.

## Results

### Cloning and Sequence Analysis of *DserNEP* Genes

Degenerate primers NEPdF and NEPdR were designed in order to amplify an internal fragment of *DserNEP* genes. These primers amplified a partial *DserNEP* sequence of 325 bp that was used as a probe to screen a *D. seriata* genomic library. Four bacteriophages containing the whole sequence of four different *DserNEP* genes were selected: phage λDASH-DsF1 contained a *DserNEP1* gene (NCBI GenBank database accession number AKQ49205), λDASH-DsF2 phage contained a *DserNEP2* gene (AKQ49206), whereas λDASH-DsF3 phage contained a *DserNEP3* gene (MK978328), and λDASH-DsF4 phage contained a *DserNEP4* gene (MK978329).

**Figure 1:**
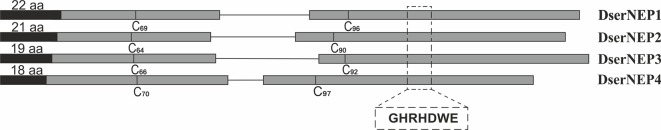
Diagram shows the overall organization of *DserNEP* genes. The E1–E2 exons are represented in dark gray boxes. Both are separated by an intron (single line). The position (relative to the initial methionine residue) of the two conserved cysteine residues typical of type I NLP proteins is indicated. Signal peptides are indicated by black boxes. The relative location of the central hepta-peptide motif typical of NLP proteins in E2 is indicated (italics).

All the *DserNEP* genes were amplified by using specific primers (**Table 1**). *DserNEP1* gene consisted of an open reading frame (ORF) of 877 bp. *DserNEP2* gene consisted of an 858 bp ORF, whereas DserNEP3 and DserNEP4 ORFs had a size of 897 and 812 bp, respectively. Total RNA isolated from *D. seriata* was subjected to RT-PCR to obtain cDNAs of *DserNEP* genes. The resulting fragments were cloned and sequenced. Sequence analysis revealed that the *DserNEP1* gene contained a 142 bp intron to yield a cDNA of 729 bp encoding a 242 amino acid protein. The *DserNEP2* gene contained a 126 bp intron to generate a 735 bp cDNA encoding a 243 amino acid protein. The *DserNEP3* gene contained a 162 bp intron to generate a 735 bp cDNA encoding a 243 amino acid protein, whereas the *DserNEP4* gene contained a 59 bp intron to generate a 753 bp cDNA encoding a 250 amino acid protein. Signal peptides were predicted by using Signal P3 software (Bendtsen et al., 2004). This analysis suggested that *DserNEP* genes contain a typical signal peptide ranging from 18 to 22 amino acids (**Figure 1**).

### Analysis of Amino Acid Sequences of *DserNEP* Proteins

According to sequence analysis, the DserNEP1 protein has an estimated isoelectric point (Ip) of 4.30 and a molecular mass of 25,384.99 Da; DserNEP2 has an estimated isoelectric point of 4.83 and a molecular mass of 25,647.41 Da; DserNEP3 protein has an estimated isoelectric point (Ip) of 5.59 and a molecular mass of 26,434.14 Da whereas DserNEP4 protein has an estimated isoelectric point (Ip) of 7.12 and a molecular mass of 27,482.45 Da.

*D. seriata* NEP proteins contain two conserved cysteine residues at positions 69 and 96 in DserNEP1, positions 64 and 90 in DserNEP2, positions 66 and 92 in DserNEP3, and positions 70 and 97 in DserNEP4. All of them also possessed the characteristic GHRHDWE central hepta-peptide motif starting at amino acid positions 132, 129, 130, and 136, respectively (**Figure 1**).

The publication of the *Diaporthe ampelina, D. seriata*, and *Phaeomoniella chlamydospora* genomes (Morales-Cruz et al., 2015), and the draft sequenced from *Eutypa lata* (Blanco-Ulate et al., 2013a), *N. parvum* (Blanco-Ulate et al., 2013b), and *Phaeoacremonium minimum* (Blanco-Ulate et al., 2013c) as well as the availability of the sequences posted at Joint Genome Institute (https://jgi.doe.gov/) revealed that there are no NLPs homologous in *P. chlamydospora* genome. *P. minimum* has two NLPs homologous (ID 1413 and 3642). *N. parvum* genome contains six NLPs homologous (ID 727, 928, 2549, 6217, 6314, and 7612). *E. lata* genome contains four NLPs homologous (ID 1995, 4041, 5290, and 8324) and *D. ampelina* has five NLPs homologous (ID 1057, 1426, 6910, 7604, and 9588), although some of them are truncated proteins.

**Figure 2:**
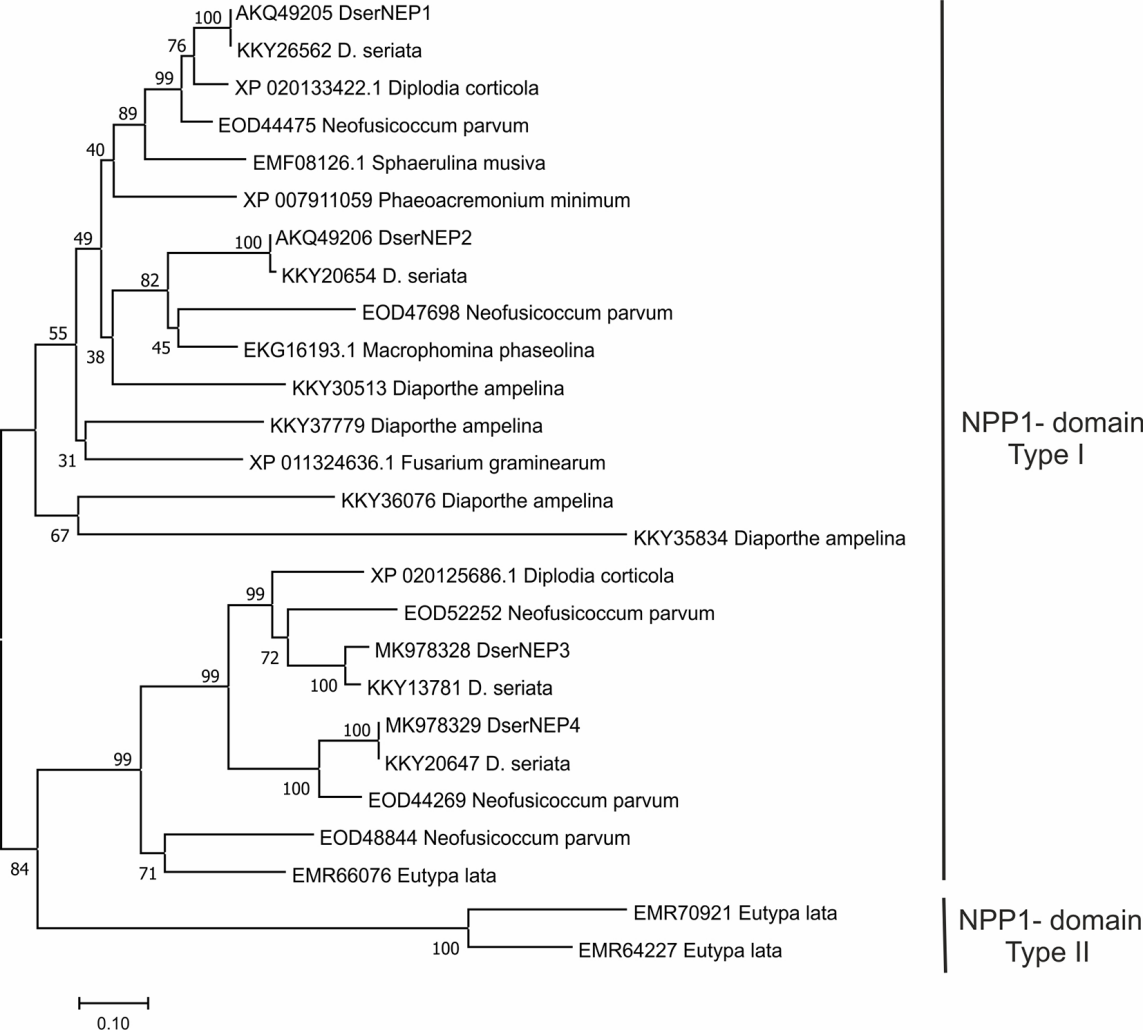
Phylogenetic tree of *D. seriata* NEP proteins with necrosis and ethylene inducing peptide 1-like proteins from GTD-related fungi. The figure shows the result of a Neighbor analysis performed using the MEGA 5.0 package (Tamura et al., 2011). Percent bootstrap values (1,000 replicates) are shown above the forks. The scale bar represents 10% weighted sequence divergence.

A comparison of the amino acid sequences from NLPs of GTD pathogens was carried out (**Figure 2**). This analysis revealed that DserNEP1 exhibited a 100% sequence identity with a putative NPP1-domain type protein of *D. seriata* (KKY26562), 90.95% with a necrosis inducing protein from *Diplodia corticola* (XP_020133422), and 79.54% with a putative NPP1-domain type protein from *N. parvum* (EOD44475). DserNEP2 exhibited a sequence identity of 99.59% with a putative NPP1-domain type protein of *D. seriata* (KKY20654), 79.15% with a npp1 domain protein (XP_020127860), and 76.13% with a hypothetical protein MPH_06630 from *Macrophomina phaseolina* (EKG16193.1), another Botryosphaeriaceae fungus, and 61.45% with a putative NPP1-domain type protein from *N. parvum* (EOD44475).

DserNEP3 exhibited a sequence identity of 100% with the hypothetical protein BK809_0004431 from *D. seriata* (OMP83050), 77.93% with a putative necrosis and ethylene inducing protein 1 precursor protein from *N. parvum* (EOD52252), and 75.00% with a necrosis and ethylene inducing peptide 1 from *D. corticola* (XP_020125686).

DserNEP4 exhibited a sequence identity of 100% with putative npp1 domain protein from *D. seriata* (KKY20647), 78.09% with a putative necrosis and ethylene inducing peptide 1 precursor protein from *N. parvum* (EOD44269), and 61.68% with necrosis and ethylene inducing peptide from *D. corticola* (XP_020125686).

### Analysis of DserNLP Expression

**Figure 3:**
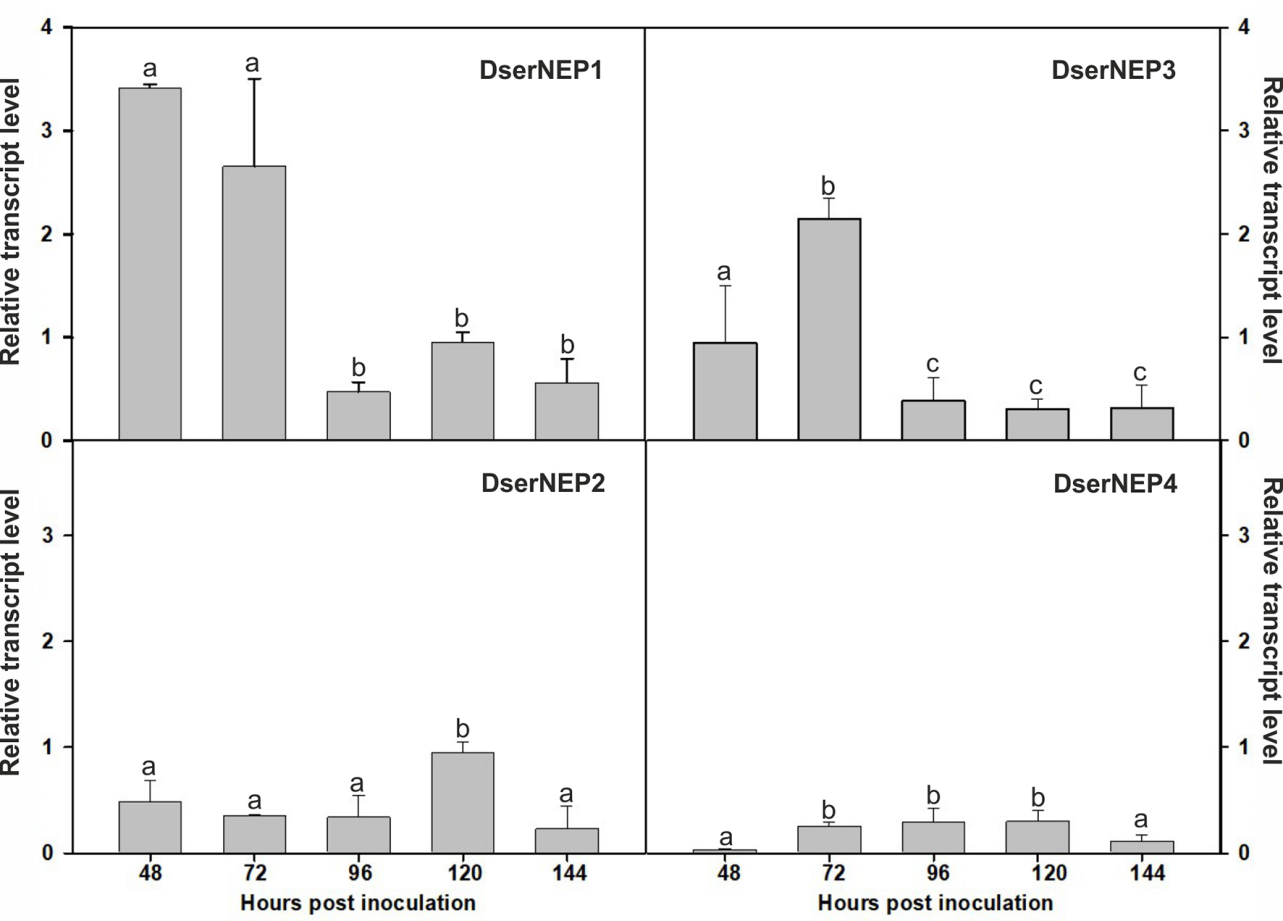
Relative transcript levels of DserNEPs. The relative transcript levels of four *DserNEP* genes were determined by q-RTPCR using β-tubulin as reference gene. Samples were recovered at 48, 72, 96, 120, and 144 hours post inoculation. Data shown represent the mean ± SD from three independent experiments. The genes analyzed were considered significantly up- or downregulated when changes in their expression were > 2-fold or < 0.5-fold, respectively. Bars marked with the same letter do not differ at P = 0.05

Expression levels of genes encoding DserNEPs in *D. seriata* VS1 strain were analyzed in Czapeck liquid medium and compared to the expression levels observed in the same medium supplemented with chips of grapevine wood from Tempranillo cultivar to mimic a putative inductor effect carried out by some component present in the wood of grapevine plants. The obtained results showed that only *DserNEP1* and *DserNEP3* genes were upregulated in the assayed conditions *DserNEP1* was induced at 48 and 72 hours post inoculation, whereas *DserNEP3* gene was induced at 72 hours post inoculation. On the contrary, *DserNEP2* and *DserNEP4* genes were downregulated in the assay conditions (**Figure 3**).

### Cytotoxic and Necrotic Activity of *D. seriata* NLPs

**Figure 4:**
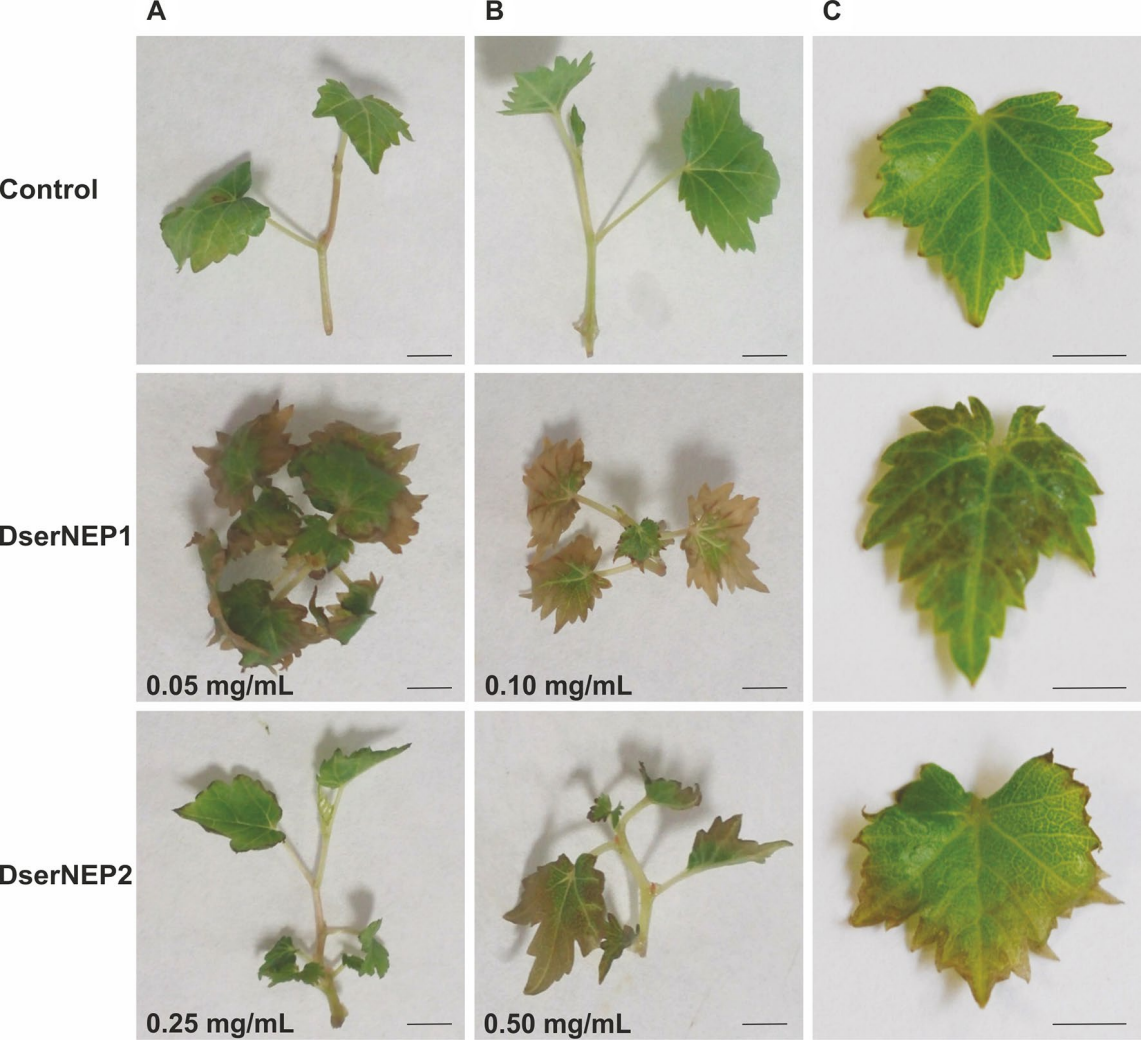
Foliar symptoms caused by DserNEP proteins. Micropropagules of *V. vinifera* were inoculated by dipping into 100 µl of different concentrations of DserNEP1 **(A)**, DserNEP2 **(B)**, or buffer **(C)** as negative control. Pictures were taken 4 days after inoculations. The experiment was repeated three times, always using three independent samples for each treatment and three negative controls (dipped into buffer). The scale bar represents 1 cm.

DserNEP1 and DserNEP2 were selected for further studies based on the fact that they had been the only two NEP proteins detected in the secretome of *D. seriata* in a previous work (Cobos et al., 2010). Genes encoding DserNEP1 and DserNEP2 proteins were cloned into the pETSUMO expression vector and expressed in *E. coli* BL21(DE3). Purified protein yields were 100 µg/mL for DserNEP1 and 500 µg/mL for DserNEP2 under the tested assay conditions. The putative phytotoxicity of NEP proteins was first determined by dipping *in vitro* micropropagated *V. vinifera* plants into NEP suspensions. We tested different protein concentrations between 0 and 0.5 mg/mL. Necrosis symptoms were clearly visible 3 days after inoculation at 0.25 and 0.5 mg/mL (**Figure 4**). At lower concentrations (0.05 and 0.1 mg/mL), only the plants inoculated with DserNEP1 showed some symptoms of necrosis. The effect caused by DserNEP2 was less than that produced by DserNEP1. In fact, a DserNEP2 concentration of at least five times higher was required for detecting some necrotic activity as compared to the effect produced by DserNEP1. The necrotic symptoms first appeared on the leaf margin and then progressed through the center of the leaves. No symptoms were evident at either the lowest protein concentration tested or in the controls.

**Figure 5:**
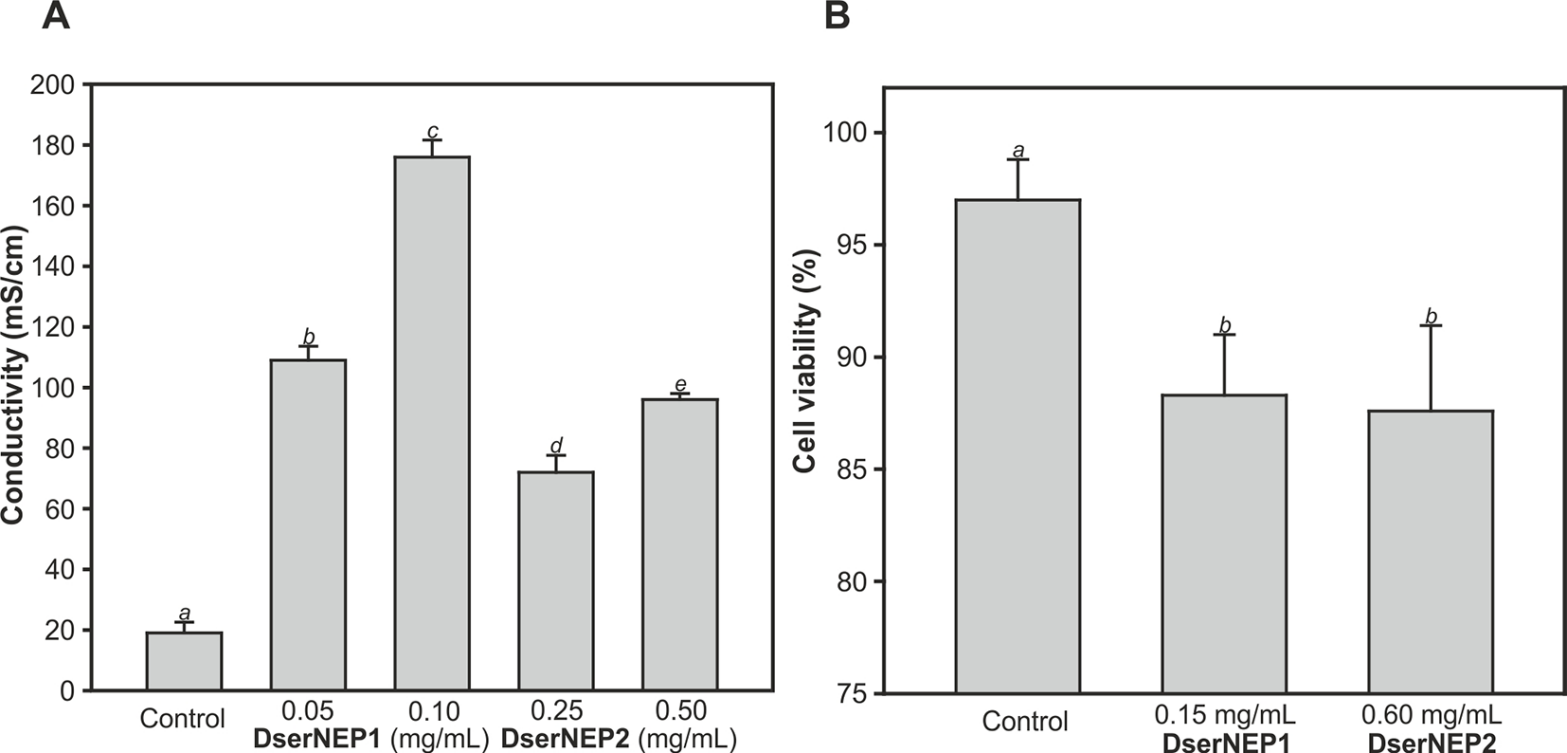
Quantification of necrotic activity by electrolyte leakage assay induced by DserNEP proteins in micropropagated plants **(A)**, and viability of *V. vinifera* cell cultures treated with DserNEP proteins determined 5 days after protein exposition by staining with FDA **(B)**. DserNEP1 was assayed at 0.05 and 0.1 mg/mL, DserNEP2 at 0.25 and 0.5 mg/mL, and control leaves were inoculated with water. Data shown represent the mean ± SD from three independent experiments. Bars marked with the same letter do not differ at P = 0.05.

The quantification of the necrotic activity was carried out by infiltration of NEP proteins into *in vitro* micropropagated *V. vinifera* plants, followed by a typical electrolyte leakage assay. We tested the same protein concentrations that we had previously used in the immersion experiments (**Figure 5A**). The conductivity data obtained for inoculated leaves was indicative of some degree of cell permeability. DserNEP1 cytolytic activity was stronger than that detected for DserNEP2; this was also the case at lower concentrations. Cell viability was tested using the FDA assay. *V. vinifera* cell suspensions were exposed to purified DserNEP1 (0.15 mg/mL) and DserNEP2 (0.60 mg/mL) proteins. Five days after inoculation, nearly all cells in the control suspensions were alive whereas cell numbers in suspensions treated with the DserNEP1 protein were slightly reduced. Up to four times higher DserNEP2 concentrations were needed for a similar response to be observed, as compared with DserNEP1 (**Figure 5B**).

**Figure 6:**
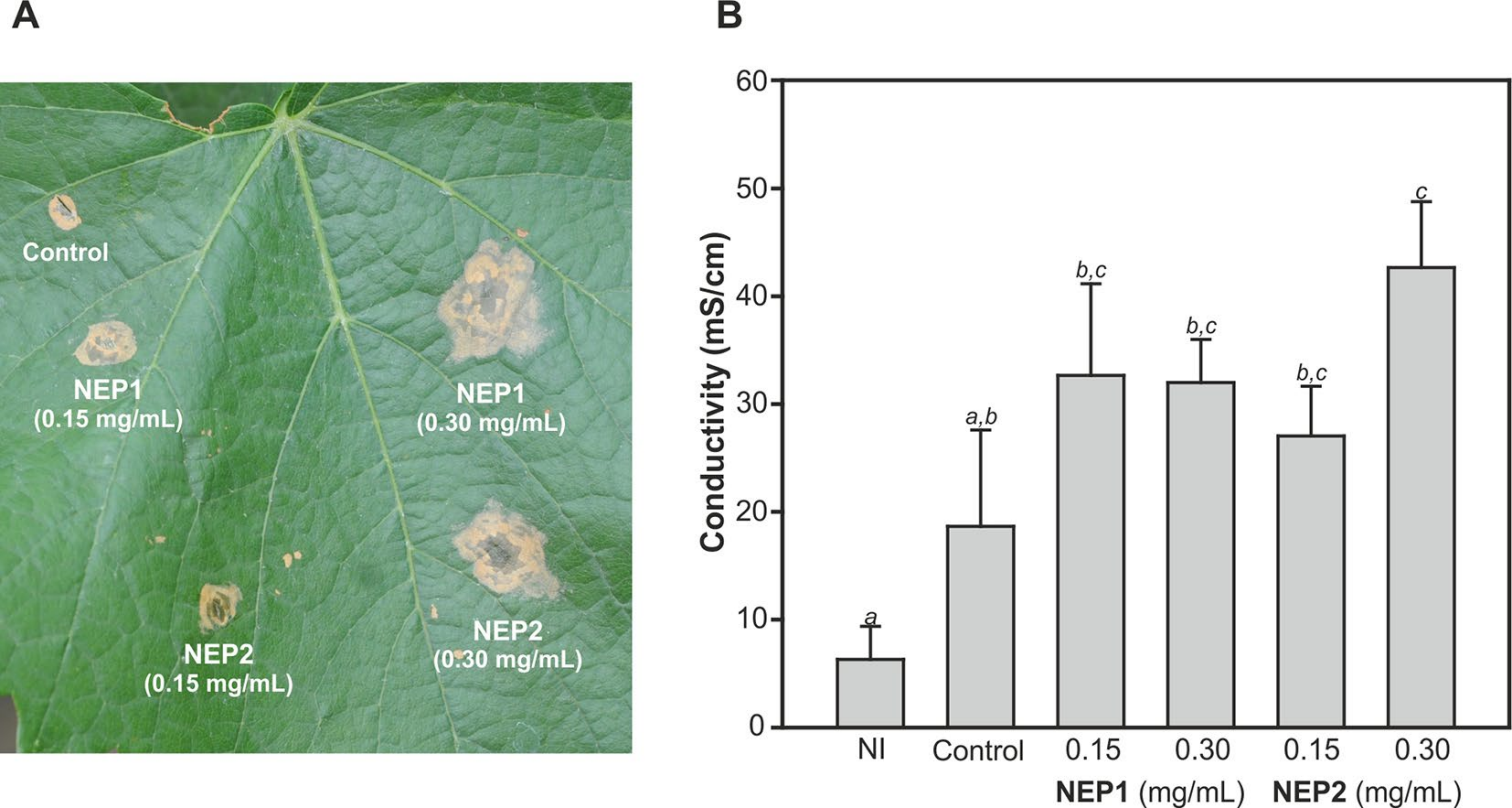
Plant necrosis promoting activity of DserNEP proteins after infiltration of leaves of 1-year-old plants. Lesion formation 4 days after infiltration is shown **(A)**. Quantification of the necrotic activity by an electrolyte leakage assay induced by DserNEP proteins. Data shown represent the mean ± SD from three independent experiments. Bars marked with the same letter do not differ at P = 0.05. Control leaves were inoculated with water. Non-inoculated leaves are marked as NI **(B)**.

In order to test the effect of these proteins on adult plants, leaves of 1-year-old Chardonnay *V. vinifera* plants were inoculated with 0.15 and 0.30 mg/mL of DserNEP1 and DserNEP2 proteins by leaf infiltration. The necrotic symptoms were clearly observed after 2 days of infiltration (**Figure 6A**). The necrotic symptoms produced by DserNEP1 were quite similar at both concentrations, and the effect was greater than the effect produced by DserNEP2 at 0.15 mg/mL (**Figure 6B**). The highest necrotic effect was produced by DserNEP2 at 0.30 mg/mL and both proteins produced larger lesions than the control (wound inoculated with buffer). Although visually the necrotic activity of DserNEP1 seemed to be slightly higher, no significant differences between the two proteins were detected by performing an electrolyte leakage assay (**Figure 6B**).

**Figure 7:**
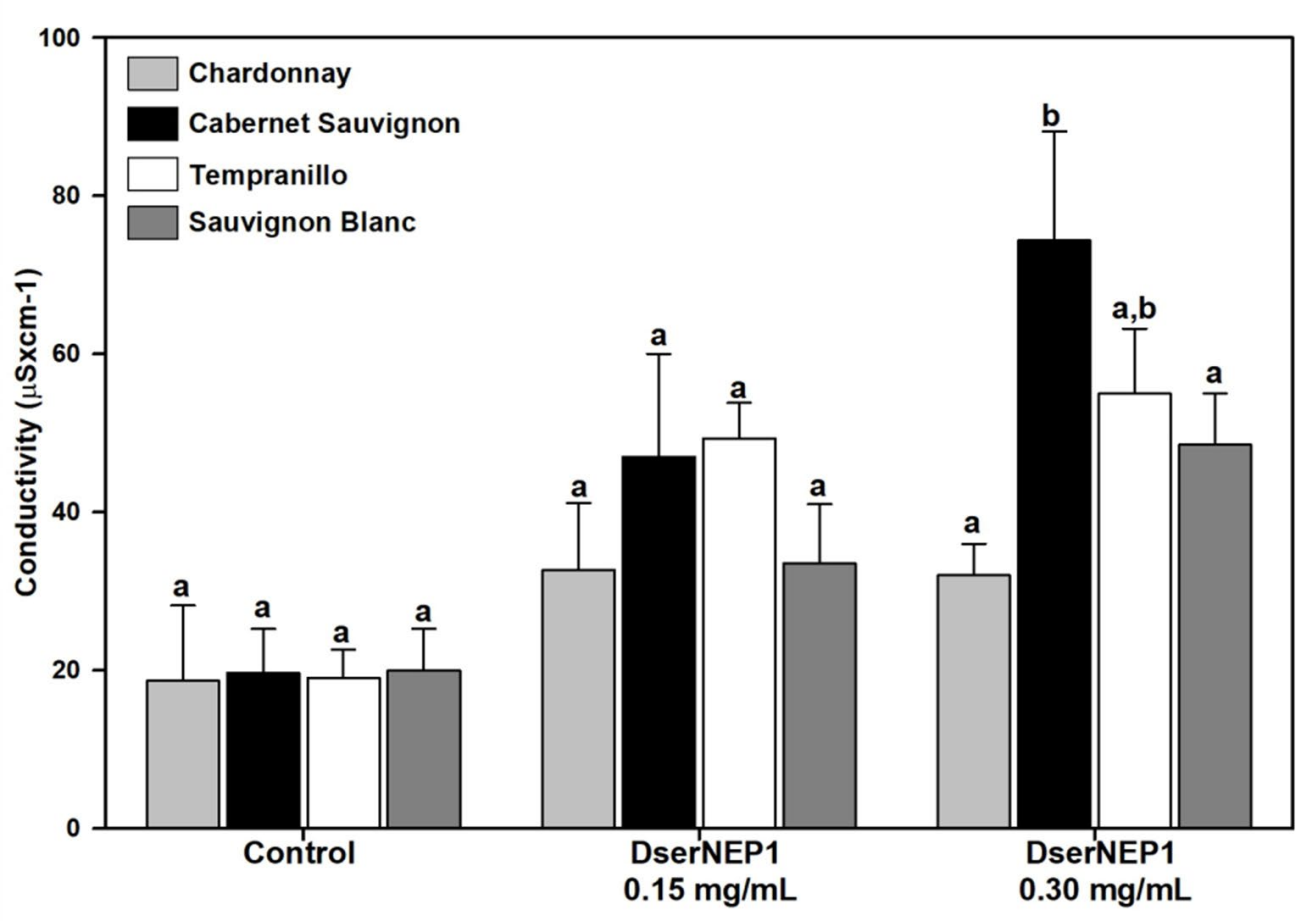
Grapevine cultivar susceptibility was tested by leaf infiltration with DserNEP1 protein. Grapevine leaves from four different cultivars (Chardonnay, Cabernet Sauvignon, Tempranillo, and Sauvignon Blanc) were inoculated with DserNEP1 at 0.15 or 0.30 mg/mL. Control leaves were inoculated with water. Electrolyte leakage was measured for 4 days after infiltration. Data shown represent the mean ± SD from three independent experiments. Bars marked with the same letter do not differ at P = 0.05.

Four different grapevine cultivars were assayed in order to test their susceptibility: Chardonnay, Cabernet Sauvignon, Tempranillo, and Sauvignon Blanc. Three plants from each cultivar and three leaves from each plant were infiltrated with DserNEP1 at 0.15 and 0.30 mg/mL, and the conductivity of the necrotic tissues was measured 4 days after infiltration (**Figure 7**). As expected, the effect caused by DserNEP1 was different depending on the cultivar tested. The largest damage was produced by DserNEP1 at 0.30 mg/mL in Cabernet Sauvignon cultivar while the smallest was achieved in the Chardonnay cultivar. Again no significant differences were found in the susceptibility of this cultivar to the two protein concentrations tested.

## Discussion

In a previous work, we had detected three hypothetical proteins in the *D. seriata* secretome with significant similarity to necrosis and ethylene inducing proteins from *Nectria haematococca* and *Sclerotinia sclerotiorum* (Cobos et al., 2010). Peptides were identified by Mascot and used to design degenerated primers in order to amplify an internal fragment of *DserNEP* genes. Two different genes were amplified from *D. seriata* encoding proteins with high sequence similarity to NLPs, DserNEP1, and DserNEP2. The progression of the *D. seriata* genome sequencing suggests the putative existence of a small family of related genes in the genome of *D. seriata* since four homologous sequences could be detected (GenBank accession numbers KKY26562, KKY20654, KKY13781, and KKY20647). Similar results had been reported for other phytopathogenic fungi such as *Botrytis* species, which have two NLPs (Staats et al., 2007b; Schouten et al., 2008); *Moniliophthora perniciosa* with three NLPs (García et al., 2007), or *Verticillium dahliae* with up to nine NLP genes (Zhou et al., 2012).

The presence of a signal peptide in their sequences is in accordance with the extracellular location of the mature proteins previously reported (Cobos et al., 2010). The conserved NPP1 domain is typical for NLP proteins (PFAM domain PF05630) (Fellbrich et al., 2002). NLPs are classified into two groups, type I and type II, depending on the presence of two or four cysteine residues at conserved positions, respectively (Gijzen and Nurnberger, 2006). Accordingly, DserNEP proteins would belong to the type I group. DserNEP proteins also possessed the characteristic GHRHDWE central hepta-peptide motif (Pemberton and Salmond, 2004). This motif is part of a negatively charged cavity exposed at the protein surface that is supposed to be important for the biological activity of NEP proteins (Ottmann et al., 2009).

The publication of the *Diaporthe ampelina*, *D. seriata*, and *Phaeomoniella chlamydospora* genomes (Morales-Cruz et al., 2015), and the draft sequenced from *Eutypa lata* (Blanco-Ulate et al., 2013a), *N. parvum* (Blanco-Ulate et al., 2013b), and *Phaeoacremonium minimum* (Blanco-Ulate et al., 2013c) have revealed that there are several NLP homologues in each fungal species, except in case of *P. chlamydospora*. Some of the detected sequences have an incomplete conserved hepta-peptide GHRHDWE motif, and sometimes they lack the upstream amino acid sequence, including the conserved cysteines, and the occurrence of premature stop codons suggests that some of these sequences could be pseudogenes (Gijzen and Nurnberger, 2006; Staats et al., 2007b).

The analysis of the amino acid sequence of DserNEPs allowed their location into two different branches of a putative phylogenetic tree, corresponding to type I (two conserved cysteines) and type II NLPs (four conserved cysteines). NLPs from GTDs pathogens belong mainly to type I group since solely *E. lata* contains NLPs from type I and type II groups. However, this analysis did not reflect the phylogenetic relationship among the fungal species checked, as it was unable to discriminate between different fungal classes. This could be an indication of an intense horizontal gene transfer between species as has been suggested by other authors (Gijzen and Nurnberger, 2006; Zaparoli et al., 2009). This hypothesis could explain the differences in G+C content observed between genes encoding DserNEP proteins and the *D. seriata* genome. While the *D. seriata* genome has a G+C content of 56.7%, this percentage is increased up to 60% in *DserNEP*1, 63% in *DserNEP*2, 64% in *DserNEP*3, and 60% in *DserNEP*4 genes, suggesting that these sequences could have been recently acquired by this microorganism and their maintenance could confer some evolutionary advantage (Pemberton and Salmond, 2004; García et al., 2007).

Little is known about how NLPs cause necrosis in plant cells, but several authors indicate that they may play as elicitors by manipulating cell death programs of the host (Fellbrich et al., 2002; Zhou et al., 2012) or acting like phytotoxins (Dong et al., 2012). The detection of DserNEPs during the fungal growth, as well as the overexpression of DserNEP1 and DserNEP3 in the presence of the wood chips, suggests a putative role of DserNEPs as virulence factors. Interestingly, in a previous work Cobos and colleagues (2010) detected that DserNEP1 and DserNEP2 proteins were upregulated in the presence of carboxymethylcellulose. These differences in the expression pattern of *D. seriata* NEP proteins could be due to the different experimental strategy used. Composition of trunk chips is much more complex than pure carboxymethylcellulose. We can speculate with the presence in trunk chips of compounds with an inducing effect on *NEP* gene expression, but we cannot rule out the presence of compounds with the opposite effect. The upregulation of NLP gene expression during infection has been described in many other plant pathogens like *Botrytis cinerea* (Arenas et al., 2010), *Magnaporthe oryzae* (Fang et al., 2017), or *Verticillium dahliae* (Zhou et al., 2012).

The necrotic activity of DserNEP1 and DserNEP2 proteins has been demonstrated. However, the differences in their toxicity could indicate differences in their mechanism of action. Both proteins can produce leaf injuries in both *in vitro* propagated and adult plants, although the effect of DserNEP1 was stronger than that detected for DserNEP2. These results are in concordance with the data of NEP activity reported by Staats et al. (2007a) in *Botrytis elliptica*. The observed differences in the results obtained from *in vitro* plants of Tempranillo cultivar and those from Chardonnay 1-year-old potted plants suggest differences in cultivar susceptibility. These different susceptibilities could be related to the specific morphological characteristics or defense mechanisms of each cultivar. Moreover, the progress of foliar symptoms was quite similar to that observed in grapevines under field conditions. This fact deserves to be highlighted.

Taken together, these results suggest a putative role of DserNEPs in pathogenesis, especially in development of the leaf symptoms observed in *D. seriata* infected grapevines. Both proteins exhibited necrotic activity although DserNEP1 produced larger lesions than DserNEP2. However, little is known about the molecular mechanisms which produce the injuries detected. *D. seriata* is a vascular pathogen, but to our knowledge it has never been isolated from grapevine leaves, suggesting that DserNEP proteins may be able to reach leaves by some unknown mechanism. The increase of conductivity detected in infiltrated leaves suggests that NLPs induce plasma membrane disruption in the host. This could drive the release of host-derived molecules that would trigger damage *via* pathogen associated molecular patterns. Other authors suggest that NLPs could interact with targets in the plasma membrane. Indeed, Schouten et al. (2008) demonstrated that NLPs are associated with membranes and are accumulated in the cytosol, nuclear membrane, and the nucleolus. They proposed that NLPs might bind to, or associate with, specific plant lectins resulting in a loss of membrane integrity, possibly through the pore-forming activity of NLPs. Intracellular accumulation of NLPs could explain the high toxicity of these proteins which act as toxins, blocking transcription, interfering with chloroplast function, or inducing programmed cell death (Bae et al., 2006).

This is the first record of Nep1-like proteins from a fungus associated with GTDs and also from a member of the Botryosphaeriaceae family. Advances made in sequencing genomes of fungi associated with GTDs have revealed the presence of NLPs in most of them. Accordingly, it might be necessary to assay the role of these proteins in the development of GTDs, and particularly in the development of foliar symptoms. Further studies of all the DserNEPs present in the *D. seriata* genome and gene replacement assays might help to elucidate their mode of action and their role in plant–pathogen interactions. Unfortunately, the difficulty in developing an efficient method for genetic transformation of *D. seriata* is hampering these studies.

## Data Availability Statement

The datasets generated for this study can be found in the Genbank: AKQ49205, AKQ49206, MK978328, MK978329.

## Author Contributions

RC and JC analyzed the data, interpreted the results, conceived and designed the experiments, and contributed materials, equipment, and analysis tools. PG-A and RC developed the grapevine *in vitro* cultures and cellular callus production. RC, CC, JÁ-P, AD-G, AI, and SG-G conducted the experiments. RC, JC, and JA wrote the manuscript. All authors reviewed the manuscript and approved the final version.

## Funding

This work was supported by Bodegas Vega Sicilia S.A. (Valbuena de Duero, Valladolid, Spain).

## Conflict of Interest

The authors declare that the research was conducted in the absence of any commercial or financial relationships that could be construed as a potential conflict of interest.
